# Most Neuroscience Data Is Not Normally Distributed: Analyzing Your Data in a Non-normal World

**DOI:** 10.1523/ENEURO.0414-25.2025

**Published:** 2026-01-02

**Authors:** Michael Malek-Ahmadi, Alexandra M. Reed, Dylan X. Guan

**Affiliations:** ^1^Banner Alzheimer’s Institute, Phoenix, Arizona 85006; ^2^Department of Biomedical Informatics, University of Arizona College of Medicine-Phoenix, Phoenix, Arizona 85004; ^3^Arizona State University, Tempe, Arizona 85281; ^4^University of Calgary, Calgary, Alberta T2N 4N1, Canada

## Abstract

While the most common statistical tests assume that the error of the dependent variable follows a normal distribution, dependent variables in translational neuroscience studies often fail to meet this assumption. Common statistical tests like the *t* test and ANOVA are based on the normality assumption, but quite often these tests are used without checking whether the dependent variable meets the normality assumption which can lead to erroneous interpretations and conclusions about observed associations. There is a significant need for the neuroscience community to utilize nonparametric statistics, particularly for regression analyses. Neuroscientists can greatly enhance the rigor of their analyses by understanding and utilizing nonparametric regression techniques that provide robust estimates of associations when data are skewed. This commentary will discuss and demonstrate analytic techniques that can be used when data do not meet the assumption of normality.

## The Normality Assumption

Estimating linear associations between numeric independent and dependent variables is staple of scientific publications, particularly in neuroscience. Linear associations measured with a correlation value or a regression coefficient provide a simple and straightforward way to interpret the data being presented; however, there are instances in which a Pearson’s correlation or linear regression estimate may not adequately or accurately estimate the association between two variables.

A major assumption of linear regression is that the error of the dependent variable follows a normal or approximately normal distribution. However, this assumption is often taken for granted in neuroscience studies and formal assessments of a dependent variable's error distribution are often not carried out prior to the analysis (Hoekstra et al., 2012). When data that significantly deviate from the normality assumption, the resulting estimates of coefficients and/or *p* values from linear regression models are likely to be biased and/or invalid (Hoekstra et al., 2012). This problem is exacerbated in studies with small sample sizes where the magnitude of associations may be influenced by the degree of skewness in the dependent variable (Hoekstra et al., 2012).

## Testing Data for the Normality Assumption

**Figure 1.**
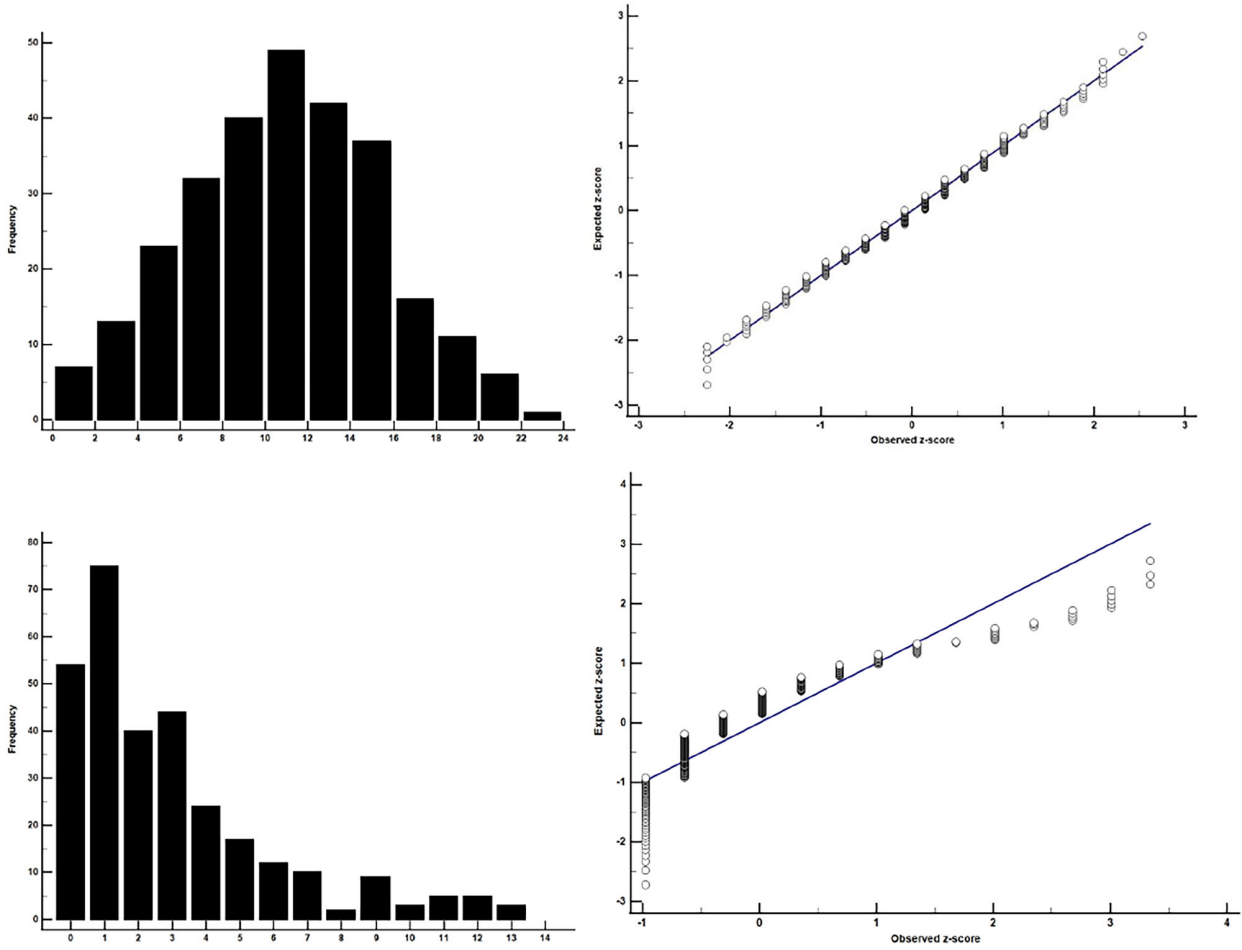
Examples of histograms and QQ plots for data that meet the assumption of normality (***A***, ***B***) and data that do not (***C***, ***D***).

The most common way to begin determining whether data meet the assumption of normality is to examine the raw data with a histogram or quantile-quantile (QQ) plot. These plots can serve as an initial check of whether a variable meets the normality assumption. Histograms can be particularly helpful as they can inform an investigator on an appropriate statistical test that can be used if there is significant skewness in the distribution. QQ plots also allow investigators to obtain a visual representation of a variable's distribution as the observed data points are plotted against values from a theoretical (normal) distribution. Data that are normally distributed will fall on a 45° reference line going from the bottom-left to the top-right of the plot. The data do not have to fall exactly on this reference line and one may notice that values at each end of the line may have noticeable deviations as these represent the tails of the distribution. Examples of histograms and QQ plots for normal and non-normal distributions are shown in Figure 1*A–D*.

There are also formal statistical tests that can be used to test the assumption of normality. The Shapiro–Wilk test is one of the most commonly used tests of normality and is available in most statistical software packages. Normality tests use *p* values where *p* < 0.05 indicates a non-normal distribution. Statistical tests for normality should be used in conjunction with histograms or QQ plots in order for an investigator to determine whether parametric or nonparametric tests should be used in the analysis. One caveat to normality tests is that they can be overly sensitive to values in the tails of a normal distribution when sample sizes are large (*n* > 500) which can result in a *p* value that is <0.05, but a histogram or QQ plot may show a normal or approximately normal distribution. It is important to note that this consideration must only be taken if an investigator is fortunate enough to work with a large sample size and given that many neuroscience datasets range from *n* = 20 to *n* = 50, a statistical test for normality should be carried out in conjunction with a visual inspection of histogram or QQ plot.

## But My Sample Size Is >30!

The central limit theorem (CLT) is a staple of introductory statistics courses in many disciplines and posits that for a given a population mean, the distribution of means from selected samples will begin to approach normality when at least 30 samples are measured. However, this theorem is often (erroneously) applied in the context of the distribution of individual data points within a sample such that it is common for investigators to employ parametric tests when their sample sizes are at least *n* = 30. While this has served as convenient rule of thumb for many decades, its misapplication to individual data points within a sample has resulted in a lack of statistical rigor for many published studies. There are many instances where sample sizes of *n* = 50 or even *n* = 100 do not yield normal error distributions for a dependent variable, yet investigators will often use parametric tests to analyze these data without checking whether the dependent variable's error is normally distributed.

While there are a number of analytic approaches that can be used with skewed data, they are not widely used by neuroscience investigators who are unlikely to have encountered these techniques in their undergraduate or graduate statistics courses. Although in an ideal world all investigators would have access to a collaborating statistician who could advise them on the appropriate analytic approach to use, this is simply not the reality we live in as most neuroscientists carry out statistical analyses on their own. This underscores the importance of bringing nonparametric techniques to the attention of investigators so that the larger neuroscience community can begin to adopt and utilize statistical approaches that will help maximize the rigor of their findings.

## But Log Transformations Can Normalize Data!

Log transformations are a common technique that many scientists use in order to “normalize” their dependent variables so that parametric tests can be used. Indeed, log transformations can be helpful in converting a skewed distribution into a normal distribution. However, there are some caveats to this that are often overlooked. Log transformations have the effect of moving the body of the distribution from left to right on the *x*-axis and can be used when the highest frequency of observations occur at the lower end of the dynamic range for a variable. This is why log transformations are often described in terms of “normalizing” data since the body of the distribution often shifts to a shape that is normal or approximately normal. When data are left skewed, or if a variable takes on a bimodal distribution, a log transformation will not result in a normal distribution. In the Methods sections of many publications, investigators often include a statement that mentions the use of a log transformation in order to normalize the data. What is often missing from these Methods sections are statements about whether the investigators checked the distribution shape prior to applying the log transformation. Likewise, it is rare that investigators report checking the distribution of log-transformed data to verify that it meets the assumption of normality.

## Nonparametric Regression

While parametric tests like the *t* test, analysis of variance (ANOVA), and Pearson’s correlation have nonparametric counterparts (e.g., Mann–Whitney, Kruskall–Wallis, and Spearman correlation, respectively), nonparametric regression approaches exist but are not widely used. Generalized linear models (GLMs) allow investigators to specify an underlying error distribution for a dependent variable used in estimating the regression model (Neuhaus and McCulloch, 2011), although there are limits to the kinds of distributions that can be specified (Neuhaus and McCulloch, 2011). In cases where a distribution cannot be specified, robust regression is another alternative that can be used (Wainer and Thissen, 1976).

Here we will discuss the rationale and application of different nonparametric regression techniques that can be used when a dependent variable does not meet the assumption of normality. While this is not an exhaustive review of the many different regression approaches that are available, we focus on ones that can be easily applied to neuroscience datasets and demonstrate how these regression approaches provide better association estimates than linear regression when data do not meet the assumption of normality.

## Robust Regression

Robust regression analysis is an approach that can be used when a dependent variable's error distribution cannot be modeled through linear regression. Although robust regression existed for several years (Wainer and Thissen, 1976; Cantoni and Ronchetti, 2006; Maronna et al., 2006), its interpretation is similar to linear regression but is not usually part of graduate-level statistics and methodology courses. The primary difference between linear and robust regression is that the former regresses individual data points using the mean while robust regression uses Maximum likelihood (M)-estimators as the regressor (Huber, 1964; Valdora and Yohai, 2014; Varin and Panagiotakos, 2019; Yang et al., 2019). M-estimators allow for valid associations to be drawn in the presence of outliers and significant skewness in a continuous dependent variable (Cantoni and Ronchetti, 2006; Malek-Ahmadi et al., 2024).

**Figure 2.**
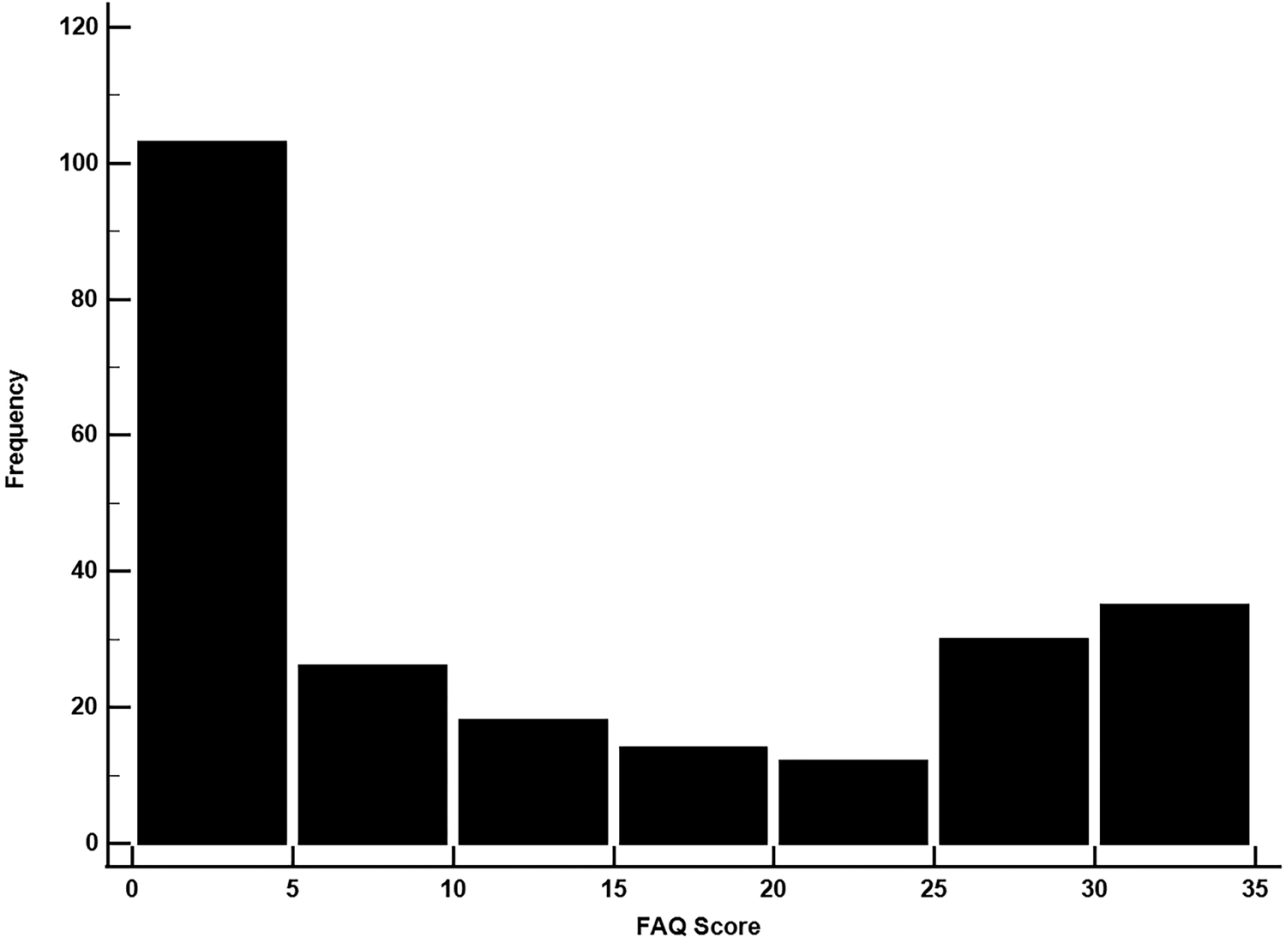
Distribution of FAQ scores is bimodal which precludes the use linear regression. A robust regression model could be used to model this variable.

To demonstrate the utility of robust regression, we will use performance on a semantic fluency test (animal fluency) to predict an individual's functional status on the Functional Activities Questionnaire (FAQ). In Figure 2 we see that the distribution of FAQ scores do not meet the assumption of normality. For this example, linear and robust regression models with FAQ as the dependent variable and semantic fluency as the independent variable will be run and their respective outputs will be compared. In Table 1 the regression coefficients are vastly different (linear model, −1.27; robust model, −0.46). While both models indicate that the associations are statistically significant, the difference in regression coefficients cannot be ignored as the linear model greatly overestimates the strength of the association when compared with the robust model. In this situation an investigator should report the results of the robust model given the non-normal, bimodal distribution of FAQ scores. If the linear model result was reported, then an overestimated effect size in a publication could lead others to (erroneously) use this estimate to determine sample sizes for their own studies. Reporting this overestimated effect size may also result in failed replications of the study.

**Table 1.** Comparison of estimates between the linear model and robust models for semantic fluency as a predictor of FAQ score

Model	*b*	95% CI	*p*
Linear regression	−1.27	−1.56, −0.98	<0.001
Robust regression	−0.46	−0.78, −0.14	0.005

Estimates were derived from two models using the FAQ as the outcome variable and semantic fluency as the predictor variable, *b* represents the coefficients from each model, FAQ, functional activities questionnaire; 95% CI, 95% confidence interval.

## Poisson and Negative Binomial Regression

Count and count-like outcomes are common in neuroscience. From spike counts in neurophysiology to commonly used clinical rating scales in clinical neuroscience, these data consistently exhibit distributional properties that may lead to biased or invalid estimates and inferences using linear regression. Specifically, these data are composed of non-negative integers that are typically right skewed and lower-bounded by zero. Count models account for these distributional properties (Nelder and Wedderburn, 1972). Poisson regression is the canonical count model; although, when variance exceeds the mean (overdispersion), negative binomial models are better suited to avoid underestimated standard errors and overly narrow confidence intervals (Ver Hoef and Boveng, 2007). Modern statistical software have made the implementation of these models increasingly trivial (Zeileis et al., 2008). However, several practical considerations are warranted to ensure that models are being applied and interpreted correctly.

**Figure 3.**
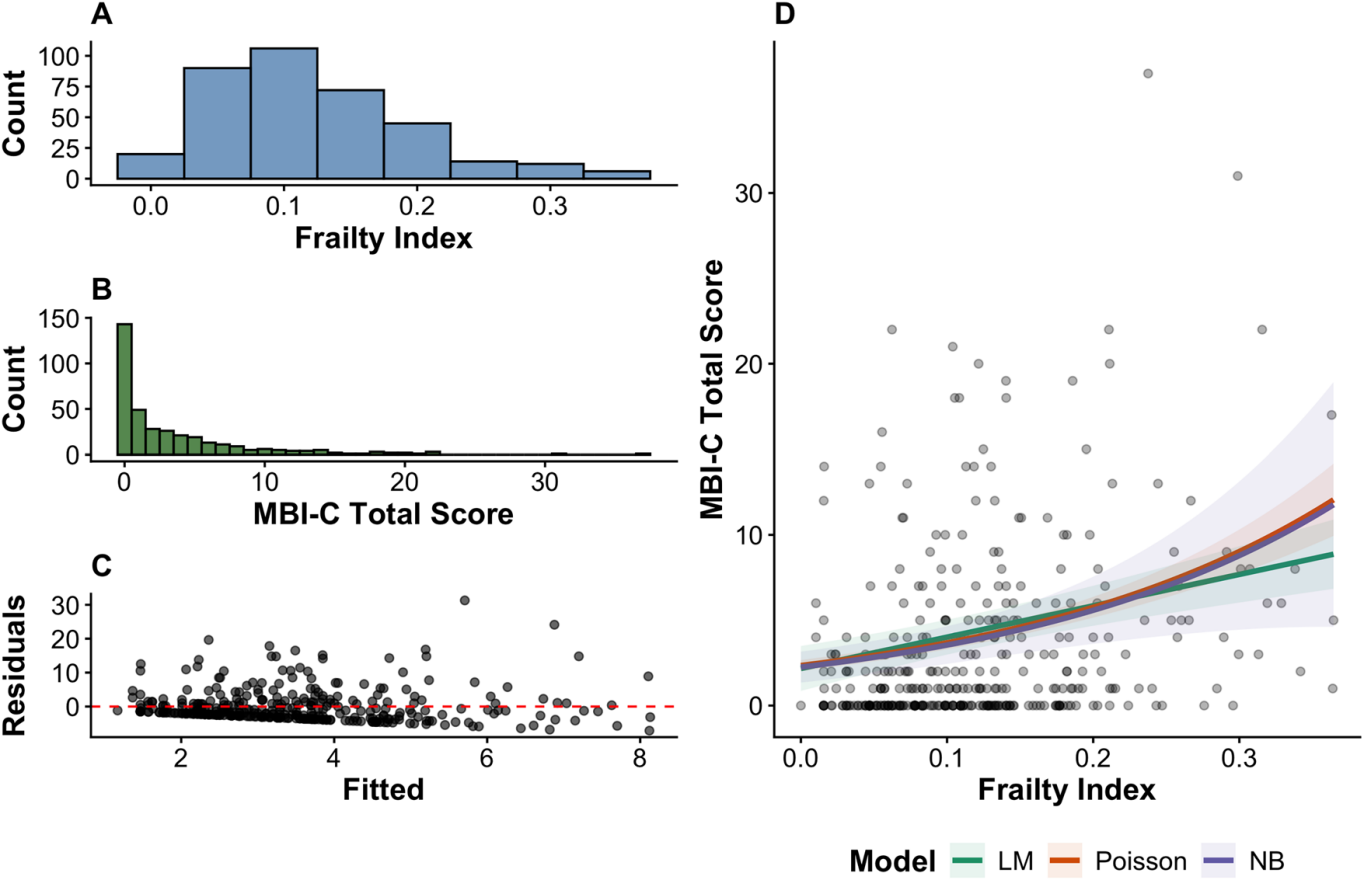
***A***, Histogram of frailty index scores across 365 participants aged ≥65 years. ***B***, Histogram of MBI-C scores across 365 participants aged ≥65 years. ***C***, Residual versus fitted plot for an unadjusted linear regression model. Asymmetric spread of data with several cases of large positive residuals suggests that the model is underpredicting several cases. ***D***, Scatterplot of MBI-C total scores as a function of the frailty index. Lines of best fit were derived from four models of MBI-C as the outcome variable and frailty index as the predictor variable, adjusting for age, sex, and years of education. 95% confidence intervals are indicated by the corresponding shaded boundaries. All four models are generally consistent at lower levels of the frailty index but begin to diverge at higher levels as the count models assume a multiplicative relationship. Confidence intervals for the linear and Poisson models fail to capture the imprecision at higher levels of the frailty index, potentially leading to false inference (e.g., Type I errors). MBI-C, mild behavioral impairment checklist; LM, linear regression model; NB, negative binomial regression model.

We provide an example of using count models for data from 365 cognitively unimpaired human participants 65 years and older from CAN-PROTECT study (Ismail et al., 2024). The predictor variable was the frailty index (FI; 0–1, often interpreted in 0.1 increments; Fig. 3*A*), a measure of overall health based on the proportion of health deficits present in a given individual (Mitnitski et al., 2001; Theou et al., 2023). The outcome variable was the Mild Behavioral Impairment Checklist (MBI-C) total score (34 items scored 0–3; range 0–104), a measure of later-life emergent and persistent neuropsychiatric symptoms (e.g., apathy, depression, impulse dyscontrol) that may signal prodromal neurological disease (Ismail et al., 2017). MBI-C data are heavily right skewed in this mostly community-dwelling sample and behaves statistically like a count, making count models an appropriate choice to analyze MBI-C data (Fig. 3*B*).

Linear regression estimated that every 0.1 increase in FI was associated with a 1.84-point higher average MBI-C total score (*b* = 1.84, 95% confidence interval [CI]: 1.12–2.55, *p* < 0.001; Table 2). However, these data clearly do not meet the normality assumption (Fig. 3*C*). Poisson and negative binomial regressions yielded multiplicative coefficients. Specifically, each 0.1 FI increment corresponded to roughly 1.5 times higher expected MBI-C total score. However, standard errors and confidence intervals were wider for negative binomial models compared with the Poisson model (Fig. 3*D*), suggesting that the negative binomial models appropriately corrected for overdispersion (Cameron and Trivedi, 1990; Kleiber and Zeileis, 2008). Furthermore, model fit indices, including Akaike information criterion (AIC; Akaike, 2003) and Bayesian information criterion (BIC; Schwarz, 1978), Vuong's test (Vuong, 1989), and DHARMa residual checks (Hartig, 2016) all favored the negative binomial over the Poisson and linear models. These data demonstrate that the MBI-C, when treated as an outcome variable, are better modeled by negative binomial regression.

**Table 2.** Comparison of estimates and model fit indices between the linear model and count models

Model	*b*	*e^b^*	95% CI	*p*	AIC	BIC
Linear regression	1.84	-	1.12, 2.55	<0.001	2,218	2,241
Poisson regression	0.45	1.54	1.47, 1.67	<0.001	2,738	2,757
Negative binomial regression	0.45	1.57	1.27, 1.96	<0.001	1,650	1,674

Estimates were derived from three models of MBI-C as the outcome variable and frailty index as the predictor variable, adjusting for age, sex, and years of education. *b* represents untransformed coefficients from each model corresponding to the frailty index predictor variable. *e^b^* represents the exponentiated count model coefficients. MBI-C, mild behavioral impairment checklist; 95% CI, 95% confidence interval; AIC, Akaike information criterion; BIC, Bayesian information criterion.

Using AIC values to compare the overall fit of different models is a practice that is becoming more prevalent. In many cases the AIC value is used to determine if the addition of variables to a model increases its predictive value. Investigators can report the results of these model comparisons in a table that specifies the variables that are included in each model along with their respective AIC values [see Janelidze et al. (2022), their Table 2 for a good example]. AIC values can also be compared between different types of regression analyses so if an investigator was uncertain about whether to use a linear or negative binomial model their respective AIC values could be compared and the model with the lowest AIC value would be the preferred model.

## GLM Gamma Regression

GLMs utilize maximum likelihood estimation (MLE), which estimates the population parameters through an iterative process, thereby finding the ones most likely to produce the observed data (Yang and De Angelis, 2013). GLMs can handle various distribution types by specifying the distribution and using a link function to relate the linear predictor to the expected value of the response variable (Coxe et al., 2013). When the dependent variable takes the form of a non-negative and nonzero real number, a GLM that uses the gamma distribution can be used to model data that are significantly right skewed.

**Figure 4.**
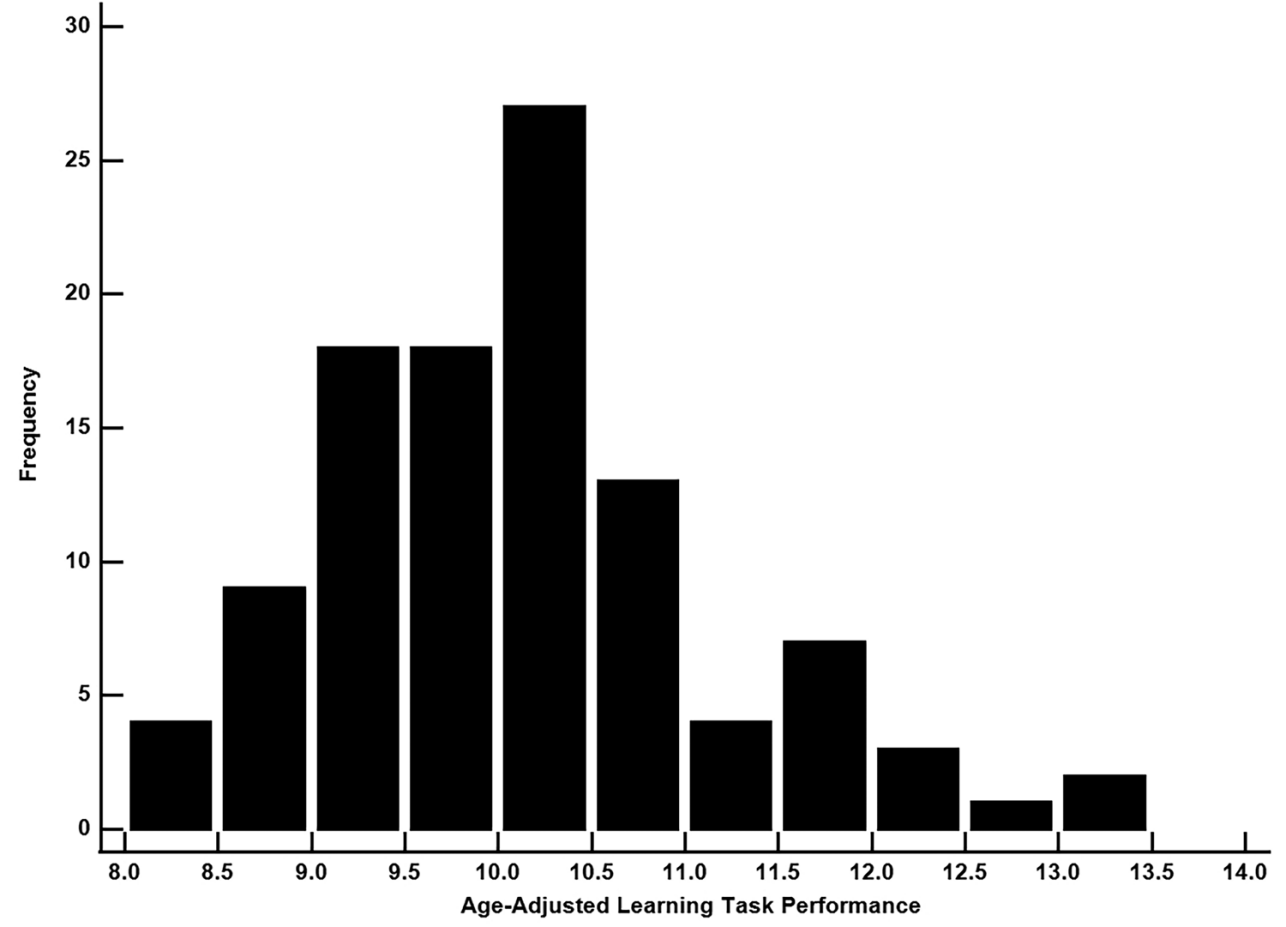
Distribution of learning task scores is right skewed with the highest frequency of scores occurring at the lower end the dynamic range. Since the numeric values are noninteger, a GLM gamma model could be used to model this variable.

In this example MRI-derived cortical thickness measurements for the entorhinal cortex will be used to predict performance on age-adjusted scores for a motor-based learning task. For the learning task, lower values indicate better performance. These data are from a cohort of older adult human participants that includes individuals who are cognitively unimpaired (CU), those with mild cognitive impairment (MCI), and those with Alzheimer's disease (AD; Malek-Ahmadi et al., 2025). In Figure 4, it is noted that there is a high frequency of scores at the lower end of the dynamic range which allows for the use of the gamma distribution in the GLM. For demonstration, both linear regression and GLM gamma models will be applied for this analysis after which the regression coefficients and model fit values (AIC and BIC) can be compared.

When the outputs of the two models are compared (Table 3), the regression coefficient for the linear model (−1.21) dwarfs the coefficient for the GLM gamma model (−0.12). A comparison of the AIC and BIC values from the two models shows that both are lower for the GLM gamma model which indicates better model fit. In this example the linear model vastly overestimates the strength of the association between learning task performance and entorhinal cortex thickness which underscores the need to understand the distribution of a dependent variable in order to apply the correct statistical technique and obtain the most accurate estimate.

**Table 3.** Comparison of estimates and model fit indices between the linear model and GLM gamma regression model

Model	*b*	95% CI	*p*	AIC	BIC
Linear regression	−1.21	−1.78, −0.63	<0.001	295	303
GLM gamma regression	−0.12	−0.17, −0.06	<0.001	289	297

Estimates were derived from two models using learning task performance as the outcome variable and entorhinal cortex thickness as the predictor variable, adjusting for age, sex, and years of education. GLM, generalized linear model; 95% CI, 95% confidence interval; AIC, Akaike information criterion; BIC, Bayesian information criterion.

## Conclusion

Here we have provided investigators with analytic approaches that can be used when the assumption of normality is not met for a dependent variable. In our continuing efforts to increase rigor and reproducibility, we must apply the same level of rigor to our statistical analyses as we do with experimental design and conduct. To address this problem, we must thoroughly evaluate the depth and breadth of statistical training that young investigators receive in their graduate programs. While it may not be feasible to put additional didactic training in statistics into a trainee's sequence of courses, there is an opportunity to enhance existing statistics courses in order for investigators to learn what to do when their data do not meet the assumption of normality. In the meantime the issues we have discussed here can be addressed through improved reporting practices in neuroscience manuscripts. In Methods sections, it has become customary to include a subsection that outlines the statistical approaches used in a manuscript and is often titled “Statistical Analysis.” Here investigators can provide greater clarity and specificity regarding how dependent variable distributions are assessed and what approaches they took properly analyze their data. Ultimately, this will result in published neuroscience studies that yield results we can all be more confident in.

## Synthesis

Reviewing Editor: Nicholas J. Priebe, The University of Texas at Austin Decisions are customarily a result of the Reviewing Editor and the peer reviewers coming together and discussing their recommendations until a consensus is reached. When revisions are invited, a fact-based synthesis statement explaining their decision and outlining what is needed to prepare a revision will be listed below. The following reviewer(s) agreed to reveal their identity: NONE.

This is a submission for the "Improving Your Neuroscience Series". It provides 3 worked examples of ways that neuroscientists can assess correlations from data that are not normally distributed.

I have read the paper carefully and I am returning a marked-up copy to the authors suggesting some revisions: inclusion of a table comparing the 3 approaches, adding a short section on diagnostics for checking normality and the quality of a regression model, and a couple of points of clarification.

Overall, I feel this is a strong contribution. Lots of common data sources in neuroscience generate non-normal data (counts, EPSP heights, spike frequencies, etc.), and applying parametric tests/models can provide seriously misleading outputs. There's a good argument to be made that the non-parametric approaches outlined in the article should be the *default*, with parametric models only applied if the authors have good evidence that they make sense. I think this paper will help bring us a bit closer to that happy day and *should* end up as a much consulted and much-cited contribution to this series.
